# AI-Enhanced Detection of Heart Murmurs: Advancing Non-Invasive Cardiovascular Diagnostics

**DOI:** 10.3390/s25061682

**Published:** 2025-03-08

**Authors:** Maria-Alexandra Zolya, Elena-Laura Popa, Cosmin Baltag, Dragoș-Vasile Bratu, Simona Coman, Sorin-Aurel Moraru

**Affiliations:** Department of Automatics and Information Technology, Transilvania University of Brasov, 500036 Brasov, Romaniadragos.bratu@unitbv.ro (D.-V.B.);

**Keywords:** heart murmurs, cardiovascular diseases, convolutional recurrent neural network, machine learning in cardiology

## Abstract

Cardiovascular diseases (CVDs) are the leading cause of death worldwide, claiming over 17 million lives annually. Early detection of conditions like heart murmurs, often indicative of heart valve abnormalities, is critical for improving patient outcomes. Traditional diagnostic methods, including physical auscultation and advanced imaging techniques, are constrained by their reliance on specialized clinical expertise, inherent procedural invasiveness, substantial financial costs, and limited accessibility, particularly in resource-limited healthcare environments. This study presents a novel convolutional recurrent neural network (CRNN) model designed for the non-invasive classification of heart murmurs. The model processes heart sound recordings using advanced pre-processing techniques such as z-score normalization, band-pass filtering, and data augmentation (Gaussian noise, time shift, and pitch shift) to enhance robustness. By combining convolutional and recurrent layers, the CRNN captures spatial and temporal features in audio data, achieving an accuracy of 90.5%, precision of 89%, and recall of 87%. These results underscore the potential of machine-learning technologies to revolutionize cardiac diagnostics by offering scalable, accessible solutions for the early detection of cardiovascular conditions. This approach paves the way for broader applications of AI in healthcare, particularly in underserved regions where traditional resources are scarce.

## 1. Introduction

In general, heart murmurs are sounds that occur during a heartbeat cycle and are accompanied by a pounding produced by turbulent blood flow near the heart [1]. CVDs include various conditions affecting the heart and blood vessels, such as coronary heart disease, strokes, and rheumatic heart disease. More than 80% of CVD deaths are caused by heart attacks and strokes, and about a third of these deaths occur in people under the age of 70 [2].

The recognition and understanding of heart murmurs has evolved extensively throughout medical history. The early detection of cardiovascular conditions, such as heart murmurs, is often important for timely intervention in improving patient outcomes [3]. René Laennec, a French physician, introduced auscultation as a method to identify heart murmurs when he invented the stethoscope in the early 19th century [4]. This innovation revolutionized cardiac diagnosis and remains an essential tool for identifying heart murmurs.

From a categorical point of view, heart murmurs may be divided into innocent or pathological types. The first class of abnormality, which is commonly found in children and adolescents, can generally be described as harmless, requiring no treatment, and can come and go throughout a child’s development. They are generally soft and have a specific sound pattern that an experienced cardiologist can identify. In contrast, pathological ones reflect some pathologies within the heart and include various abnormalities such as valvular heart diseases, septal defects, and heart valve regurgitation [5]. Valve-related diseases of the heart include aortic stenosis and mitral valve prolapse, where the opening or closing of the heart valves is improper, affecting the flow of blood [6].

Septal defects include conditions such as ventricular or atrial septal defects that create abnormal openings in the wall of the heart between the left and right sides, allowing blood to flow between them. Heart valve regurgitation involves an improper closure of the valves, which allows blood flow. These conditions underscore the importance of accurate diagnosis and management of heart murmurs to prevent further cardiovascular complications [7].

The most common method of diagnosing heart murmurs is with a stethoscope [8] during a physical examination. Other diagnostic tests include echocardiogram, electrocardiogram (ECG), chest X-ray, or cardiac magnetic resonance imaging (MRI).

Physical auscultation is variable among observers in its accuracy. Advanced imaging techniques are, indeed, informative but costly and time-consuming. As a consequence, these challenges highlight the pressing need for more efficient, accessible, and accurate diagnostic methods, such as those enabled by advances in artificial intelligence, more precisely machine learning. These technologies can enhance traditional diagnostic methods by improving the rates of heart murmur detection and accuracy in interpreting their significance, leading to better patient outcomes [9].

**Figure 1:**
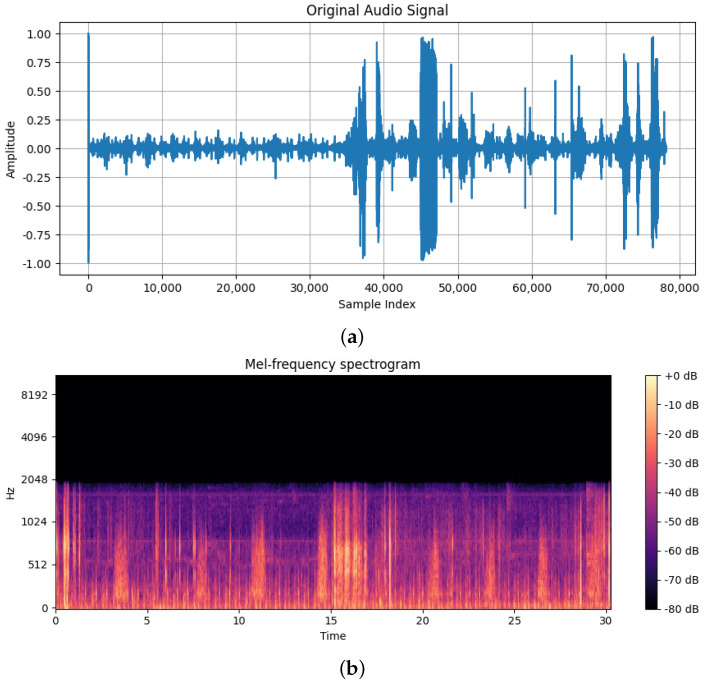
(**a**) The original sound wave; (**b**) The associated spectogram.

Artificial intelligence (AI), especially machine learning (ML), has begun to transform heart murmur detection by automating the analysis of heart sound recordings [10,11]. Artificial neural networks, which are also capable of distinguishing subtle patterns in audio data, offer a quantum leap in diagnostic accuracy and reliability. These technologies allow continuous improvements in diagnostic tools, which could reduce the rate of diagnostic errors and enhance early detection capabilities [12]. The first step in developing such a model is constituted by a rigorous analysis of the original wave sound. In Figure 1, an example of how analysis between the original one and the associated spectogram can be seen.

Machine learning can analyze vast amounts of cardiac audio data with precision for subtle patterns that may not be audible by the human ear. More precisely, these can use CNNs and RNNs to process time-series audio data from heart sounds to identify and classify murmurs with high accuracy [13].

This technology not only improves diagnostic accuracy but also reduces reliance on specialized medical staff and equipment. Additionally, AI models can be trained using diverse patient data, making them more adaptable and effective in different clinical environments. By leveraging these advancements, cardiac care can become more accessible and efficient, particularly in regions where traditional diagnostic tools are unavailable due to limited resources [14].

From an architectural point of view, the structure of the paper is divided into three main chapters. It begins with an introductory chapter that provides historical context, outlining the longstanding challenges in cardiac diagnostics and the need for innovative solutions. This sets the stage for explaining how advancements in artificial intelligence can potentially address the limitations of traditional diagnostic methods. The second chapter then delves into the technical details of the study, describing the data collection process and the data pre-processing techniques applied. It also outlines the development of the convolutional recurrent neural network (CRNN) model, including the model architecture and the iterative refinements made. Finally, the paper culminates in a chapter that presents and analyzes the performance results of the CRNN model, attributing the impressive metrics to the specific methodological choices. This structured approach allows the authors to guide the reader from the historical background, through the technical specifics, and ultimately to the impactful outcomes of the study, facilitating a clear understanding of the researchers’ work and the significance of their contributions to advancing non-invasive heart murmur detection.

## 2. Methods

To attain the objectives set forth in this study, a series of essential steps were undertaken. First and foremost, a robust dataset was collected, which would enable the development and testing of the model. The next step involved the preprocessing of these data, to bring them to an optimal format and quality. Only after completing these preparatory stages was it possible to proceed with the construction and training of the CRNN model itself.

Each of these steps played a very important role in the ultimate success of the research. In the following, the details of how each aspect of the methodology was approached, from data collection to the optimization of the CRNN model architecture, will be investigated.

### 2.1. Data Collection

**Figure 2:**
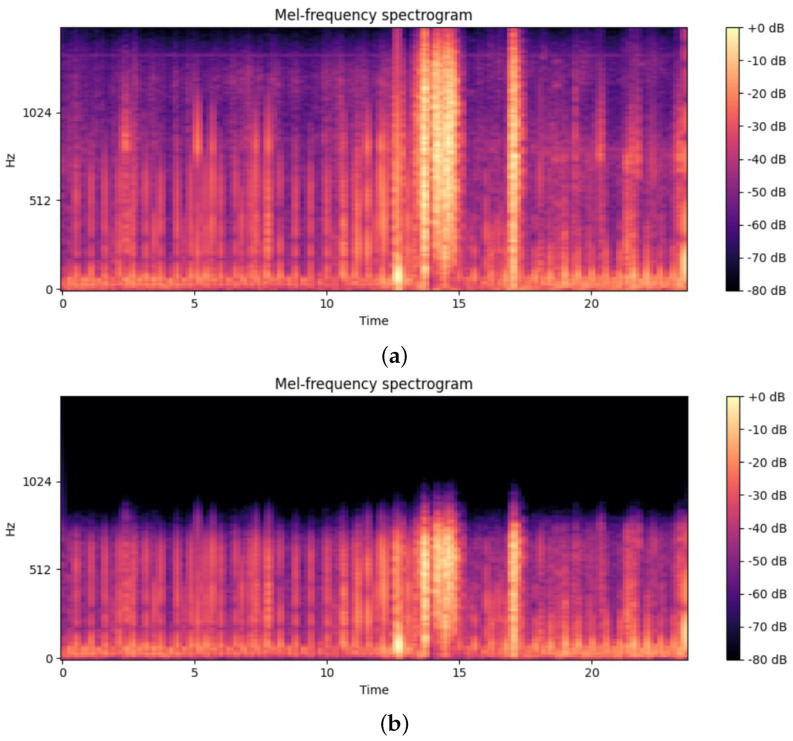
(**a**) The original recording; (**b**) The recording after pre-processing stage.

For this research, the CirCor DigiScope Phonocardiogram open source Dataset v1.0.3 was chosen because of its extensive and detailed coverage of pediatric cardiac conditions. Sixty percent of the dataset was used for training, while the remaining 40% was reserved for validation and testing. The dataset was collected during two large-scale screening campaigns in northeast Brazil, focusing on children, who are a crucial group for studying congenital and acquired heart diseases. This included 5272 heart sound recordings, totaling over 33.5 h, that resulted in 3165 recordings from 942 patients for the training set and the rest for validation, from four main auscultation sites. Out of 3165 available recordings, only 2000 were used for training. This included 1500 recordings without murmurs (negative) and 500 with murmurs (positive), while the rest were labeled as “unknown”. Each recording is richly annotated, providing demographic details as well as specific information about murmurs, such as their type, location, timing, shape, pitch, grade, and quality. These detailed annotations make the dataset an essential resource for developing and testing automated heart murmur detection methods. An example of a sample can be seen in Figure 2.

Other data, like sex, age, height, weight, and pregnancy status, greatly increases the robustness and specificity of algorithms designed for the detection of heart murmurs when incorporated together with heart sound recordings [15]. Sex, age, pregnancy status, height, and weight all play key roles in diagnosing cardiovascular conditions and murmurs. Biological sex influences conditions like mitral valve prolapse, which is more common in females, while age helps distinguish between innocent pediatric murmurs and concerning adult murmurs. Pregnancy can cause changes in the cardiovascular system that may result in new murmurs, so knowing the patient’s pregnancy status aids in distinguishing normal changes from potential heart problems. Additionally, height and weight affect heart size and sound intensity, with thicker chest walls in obese patients potentially making murmurs harder to detect, thus requiring these factors to improve diagnostic accuracy.

This analysis, combined with demographic information, provides valuable context for acoustic data. It helps AI-driven diagnostic tools achieve better predictive accuracy, reliability, and performance across different patient groups.

Data augmentation is an essential technique in machine learning, especially in the context of unbalanced or not large enough datasets that would train robust models. This approach is of particular importance in audio processing for heart murmur detection due to the natural imbalance between recordings with murmurs and those without. Most of the initial dataset is of normal heart sounds, so augmentation helps balance the dataset and improve the generalization capability of the model [16].

**Figure 3:**
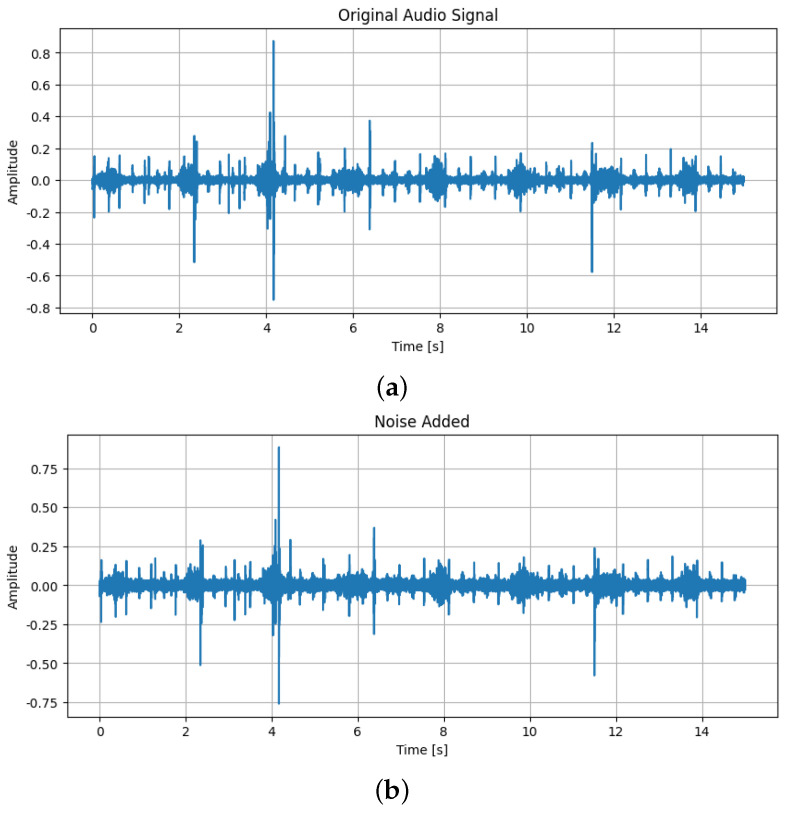
(**a**) The original sound; (**b**) The sound after Gaussian noise was added.

**Figure 4:**
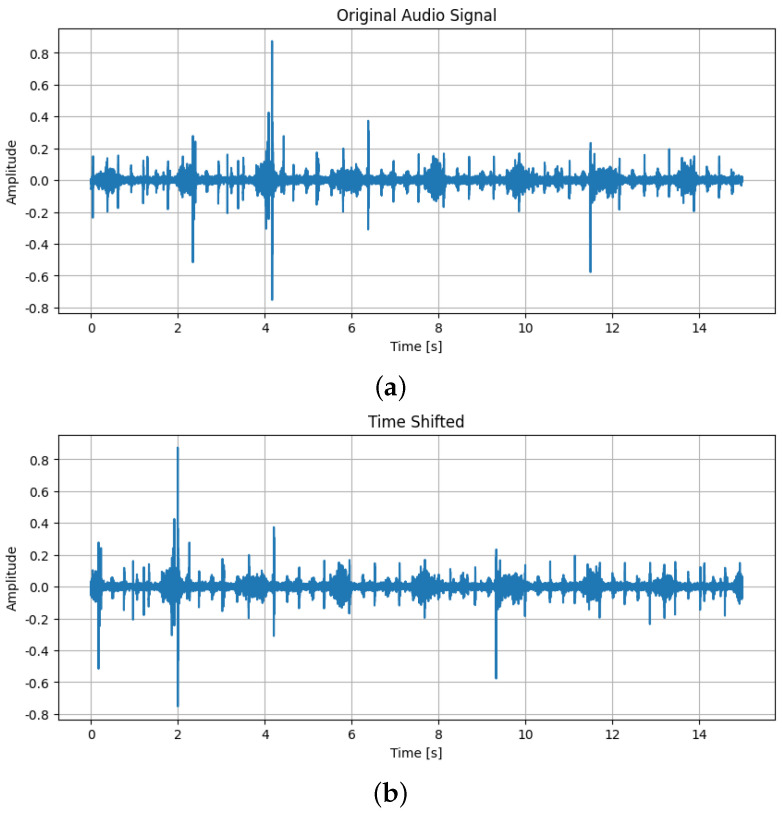
(**a**) The original sound; (**b**) The sound after time shift.

**Figure 5:**
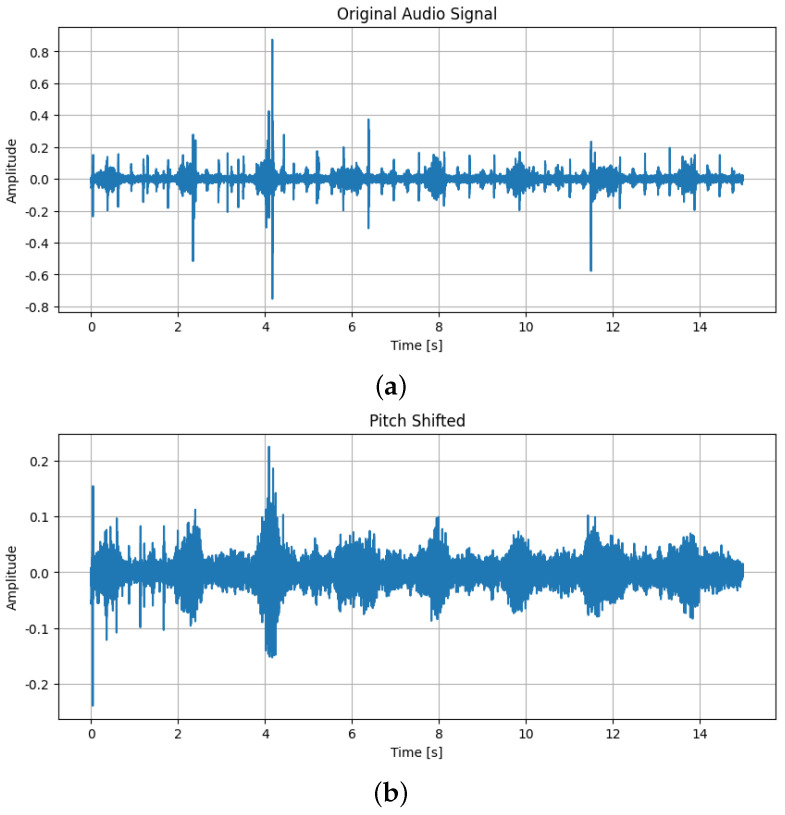
(**a**) The original sound; (**b**) The sound after pitch shift.

Audio augmentation techniques that were used are as follows:

- Addition of Gaussian noise, which is characterized by a bell-shaped probability density function following a normal distribution. When added to the original audio, it simulates real-world variability and enhances the robustness of detection models [17,18]. By overlaying the original heart sound with random noise from a Gaussian distribution, this technique helps the model focus on the key characteristics of murmurs while learning to ignore irrelevant sounds. The difference between the original sound and the same one after Gaussian noise was added can be seen in Figure 3;
- Applying time shifting, a technique where the audio signal is shifted along the time axis without altering its length and creates temporal variations in the signal. This helps train the model to recognize murmurs regardless of when they occur in the audio clip [19]. It is particularly useful for handling cases where heart murmurs appear at different times in various recordings. The difference between the original sound and the same one after time shift can be seen in Figure 4;
- Applying a pitch-shifting technique that alters the pitch of the audio signal, thereby changing the frequency characteristics of the heart sound. This creates variations in pitch, allowing the model to learn murmur representations across a wide frequency range. As a result, the model can better generalize and detect murmurs in patients with different pitches, ages, sizes, and heart conditions [20]. The difference between the original sound and the same one after adding pitch shift can be seen in Figure 5;

These augmentation techniques are essential for addressing the imbalance in the dataset and the variability inherent in audio. By artificially expanding the dataset, the model becomes better equipped to handle diverse and challenging diagnostic scenarios, ultimately improving its accuracy and reliability in clinical applications. Is is relevant to mention that all the techniques were applied to each model.

### 2.2. Data Pre-Processing

Pre-processing the dataset for heart murmur detection is important for ensuring data consistency and improving the quality of the data used for training and testing the machine-learning model.

The following steps were made in order to prepare the data:

- The sampling rate was set to 4000 Hz to capture the heart sound frequency range. This strategic choice balances capturing the necessary frequency components and managing data size and processing requirements. Heart sounds typically range from 20 Hz to 1000 Hz, and a sampling rate of 4000 Hz ensures these frequencies are well-represented without aliasing, adhering to the Nyquist theorem [21].

This high sampling rate allows for precise temporal resolution, important for accurately capturing the rapid changes in heart sounds enhancing the quality of extracted features, such as the timing and amplitude of heartbeats, which are essential for detecting abnormalities like murmurs. Additionally, a higher sampling rate can improve the performance of advanced signal processing techniques by providing more detailed data. While higher sampling rates increase the amount of data to be processed and stored, this rate strikes a good balance between capturing detailed heart sound information and managing practical constraints. This approach is also utilized in other studies, as demonstrated in works such as [22,23].

**Figure 6:**
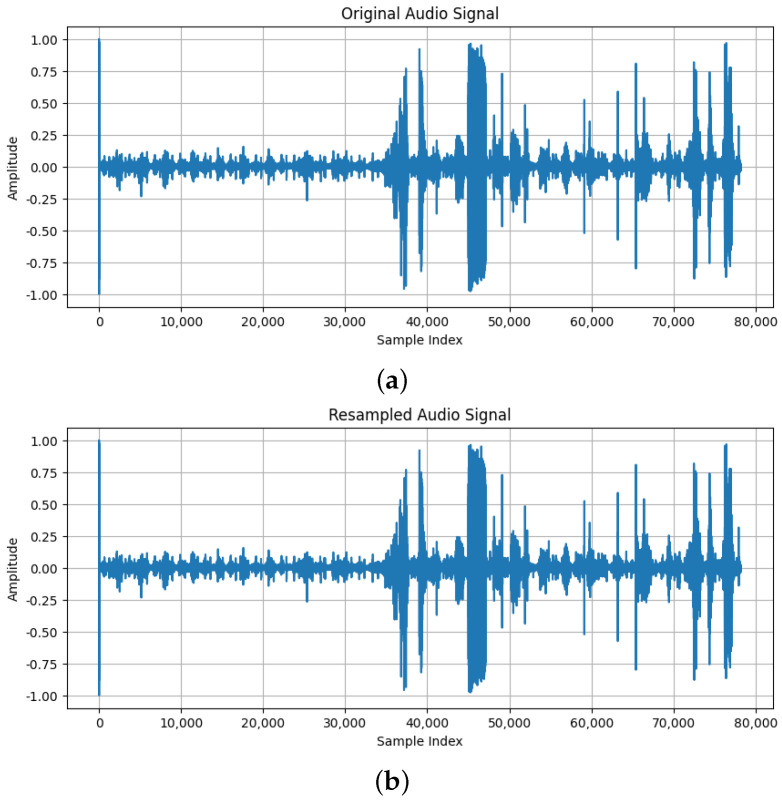
(**a**) The original sound; (**b**) The sound after standardization of sampling rate.

Standardizing the rate ensures consistent data processing and improves feature extraction and comparability across recordings Hz. The difference between the original sound and the same one after standardizing the sampling rate can be seen in Figure 6.

**Figure 7:**
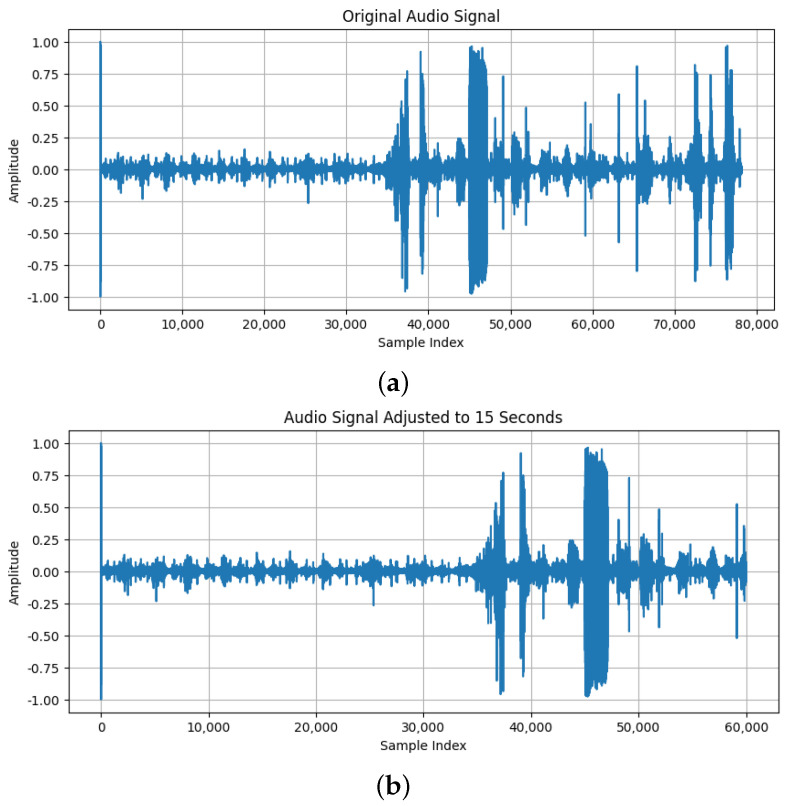
(**a**) The original sound; (**b**) The sound after normalization of recording length (15 s).

**Figure 8:**
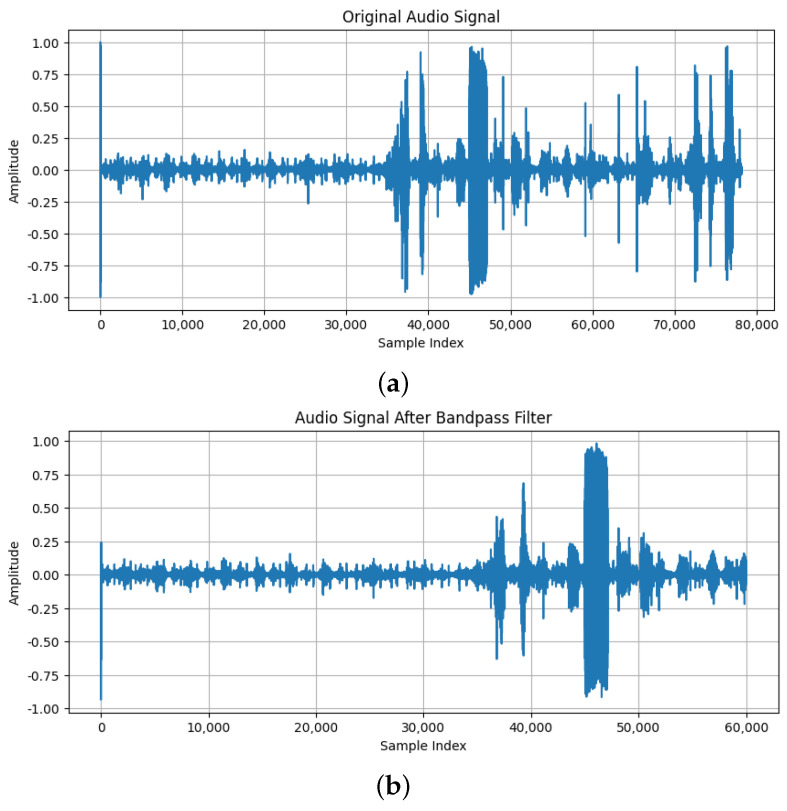
(**a**) The original sound; (**b**) The sound after band-pass filter was applied.

**Figure 9:**
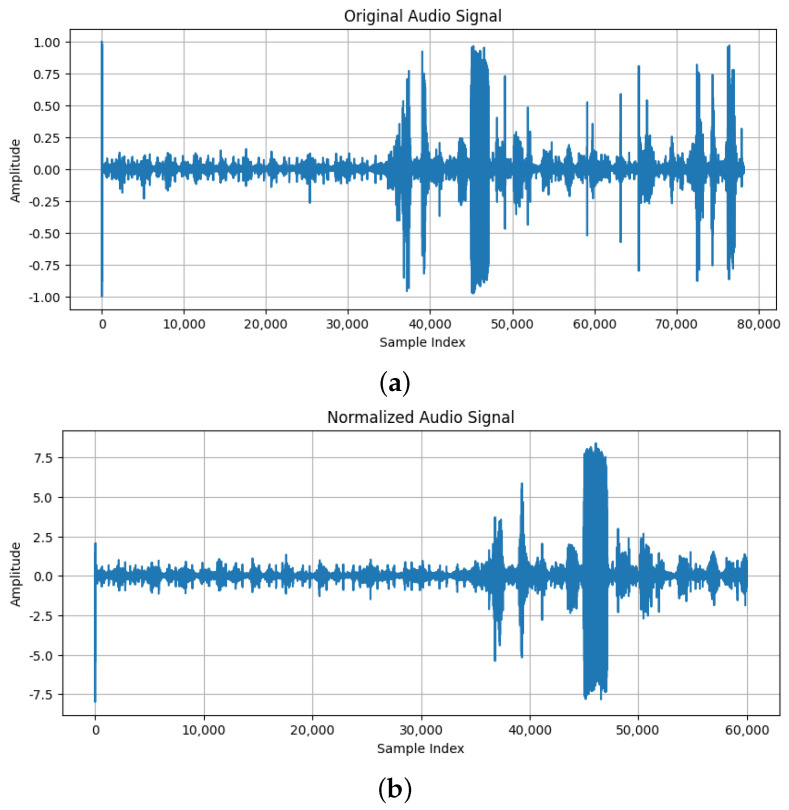
(**a**) The original sound; (**b**) The sound after Z-score normalization.

- All recordings were normalized to a uniform length of 15 s. A 15 s recording is generally sufficient to capture multiple cardiac cycles, including systole and diastole phases, providing a comprehensive snapshot of the heart’s activity. In clinical practice, physicians typically listen to heart sounds for a few seconds to detect abnormalities, and a 15 s recording aligns with this practice, providing enough time to capture relevant heart sound events without overwhelming the clinician with excessive data. Longer recordings were truncated, and shorter ones were extended with zero-padding. This ensures that each entry contributes equally to the model, preventing recording length from affecting performance. The difference between the original sound and the same one after the normalization of recording length can be seen in Figure 7.
- Band-pass filtering was applied to focus on frequencies between 20 Hz and 1000 Hz, which are most indicative of cardiac murmurs, while attenuating other frequencies. This filtering helps minimize irrelevant noise and enhances the clarity of heart sounds, improving the accuracy of murmur detection [24]. The difference between the original sound and the same one after adding band-pass filtering can be seen in Figure 8.
- Z-score normalization was applied to all audio signals, making the mean zero and the standard deviation one. This process ensures that the model is not biased by the scale of the data. In cardiac murmur detection, it prevents amplitude variations in the recordings from negatively impacting the model’s learning capability [25]. The difference between the original sound and the same one after applying z-score normalization can be seen in Figure 9.

#### Data Distribution

The dataset was divided into training, validation, and testing subsets to ensure a thorough evaluation of the model’s performance and its ability to generalize to new data. Each subset has a specific role:

- Training Data: This subset is used to train the model, helping it learn and update its parameters;
- Validation Data: This subset is used during the development process to evaluate the model’s performance. It plays a key role in fine-tuning hyperparameters, preventing overfitting, and providing an ongoing check on the model’s progress throughout training;
- Testing Data: This subset is reserved for evaluating the final model after training. It provides a fresh set of examples to see how well the model can generalize to new, unseen data.

The dataset is split into 80% for training and 10% each for validation and testing. This balance ensures the model has enough data to learn, while still having enough data to check its performance fairly and accurately.

### 2.3. CRNN Architectural Design and Conception Methodology

The CRNN architecture combines convolutional neural networks (CNNs) for spatial feature extraction and recurrent neural networks (RNNs) for temporal processing, as described in [26,27,28]. This makes it ideal for tasks that require both spatial and temporal analysis, such as analyzing audio signals for cardiac murmurs.

Convolutional recurrent neural networks offer a powerful combination of convolutional neural networks (CNNs) and recurrent neural networks (RNNs), making them particularly effective for analyzing heart murmurs. CNN layers in CRNNs automatically extract spatial features from heart sound signals, capturing essential patterns, while RNN layers handle sequential data, making them ideal for modeling the temporal dependencies in heart sound signals. This combination leads to improved accuracy in detecting heart murmurs compared to traditional machine-learning models and standalone CNNs. Additionally, CRNNs can distinguish between relevant heart sound features and background noise, enhancing robustness. Their end-to-end learning capability allows for better integration of feature extraction and classification, leading to more accurate and efficient analysis. CRNNs are also scalable, adaptable to different datasets, and can incorporate attention mechanisms to highlight relevant parts of the heart sound signal, providing insights into the decision-making process of the model. Overall, CRNNs offer a comprehensive and effective approach to heart murmur detection, making them a valuable tool in this domain.

After more experimental architecture, the one that had the best outcome is presented below:

- Layer 1: 16 filters, size (3 × 3), stride 1, padding 1, followed by rectified linear unit (ReLU) activation—the weights are initialized using the Kaiming method to optimize the behavior of ReLU.;
- Layer 2: 32 filters, size (3 × 3), stride 2, padding 1, continue with ReLU activation;
- Layer 3: Further expands to 64 filters, size 3 × 3, with a stride of 2 and padding of 1, and again uses ReLU for activation. These layers are done in succession to increase the network’s capacity for recognizing progressively more complex features from the input spectrograms of heart sounds by adding more depth to the feature detection capability.

The functionality of the architectural layers are presented below:

- Adaptive Pooling: Normalizes output sizes across different inputs, preparing the data for sequential processing;
- Dropout: Set at a rate of 0.5, it helps prevent overfitting by randomly deactivating a fraction of neurons during training;
- Recurrent Layer: Utilizes RNN structures (possibly LSTM or GRU) to capture temporal dependencies and analyze time-based patterns in heart sound signals;
- Fully Connected Layer: Combines spatial and temporal features into a comprehensive output, typically using a sigmoid function for binary classification to distinguish between normal and murmur-laden heart sounds.

The raw audio signals are first converted into spectrograms or similar forms, which then pass through convolutional layers to extract spatial features. These features are followed by recurrent layers that capture the temporal relationships.

The entire network is trained in one go, using a loss function like cross-entropy to improve classification accuracy. This approach helps the model learn from both the spatial and temporal aspects of the data. By analyzing frequency and sequence information together, the CRNN model is particularly effective for detecting cardiac murmurs.

The CRNN architecture is powerful in identifying complex patterns in heart sound data, leading to better diagnostic accuracy and early detection. It outperforms previous methods by combining detailed texture from heart sound spectrograms with temporal patterns that indicate potential health issues [29].

**Figure 10:**
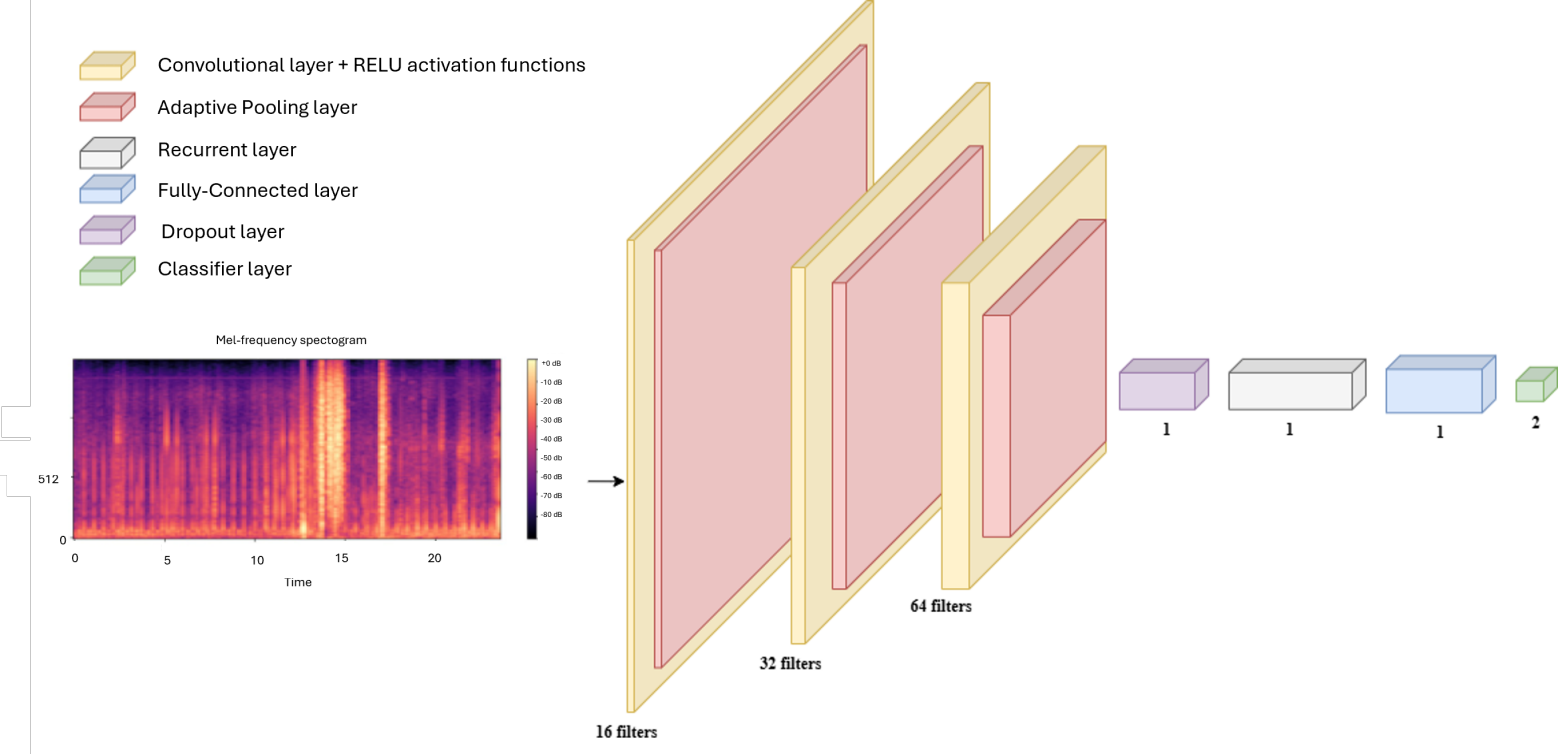
The proposed CRNN architecture.

The architecture is shown in Figure 10 and is tipped with one or more fully connected layers that integrate the high-level features extracted by the CNN and then processed by the RNN for a final decision. The output layer takes a softmax activation function—for multi-class classification problems—or a sigmoid activation function for binary classification problems.

The CRNN architecture is an effective way to combine the details in heart sound spectrograms with the timing patterns that help differentiate between normal and abnormal sounds. In practice, raw audio signals are first converted into spectrograms or time-frequency representations. These are then processed through the CRNN, where the convolutional layers extract important features, and the recurrent layers capture the timing patterns of the heart sounds.

The CRNN model learns from all parts of the data in one go, optimizing performance by minimizing a loss function like cross-entropy for classification tasks. This approach gives a powerful solution for automatically analyzing heart sounds, as it can detect complex patterns in the data. By learning from both the spatial and temporal aspects of the heart sounds, the CRNN model offers better generalization than traditional methods. This makes it more accurate for diagnosing heart conditions and helps detect cardiac murmurs early, which is crucial for timely and effective treatment.

In summary, the CRNN architecture is a powerful method for analyzing audio signals, as it can process both frequency and timing information at the same time. This makes it especially useful for detecting cardiac murmurs in clinical practice. It is an effective solution for automatically analyzing heart sounds, and is capable of recognizing complex patterns in the data. Learning both the spatial and temporal aspects of heart sounds, the CRNN model outperforms traditional methods. This leads to better diagnostic accuracy and helps detect cardiac murmurs early, which is critical for providing timely and effective treatment.

## 3. Results

In order to achieve satisfactory results for the final model, several configurations were tested by changing the number of neurons, layers, and overall architecture. A summary of all results for each model can be seen in Table 1. The evolution of metric values, including the area under the ROC curve (AUC), can be noticed through the analysis of the last model, which is the proposed one.

### 3.1. Baseline Model

The implemented CNN model consisted of three convolutional layers designed to extract features from the audio data for detecting cardiac murmurs. A significant challenge in the dataset was the imbalance, with 493 instances labeled as ‘murmur present’ and 1909 labeled as ‘murmur absent’. This imbalance likely caused the model to favor the majority class, negatively impacting its diagnostic performance.

No data augmentation techniques were applied, limiting the model’s exposure to a broader range of variations in the data. This restriction likely hindered the model’s ability to generalize effectively to unseen data. Additionally, the training process did not incorporate dynamic adjustments for the learning rate or early stopping, both of which are essential for preventing overfitting and ensuring efficient convergence.

**Figure 11:**
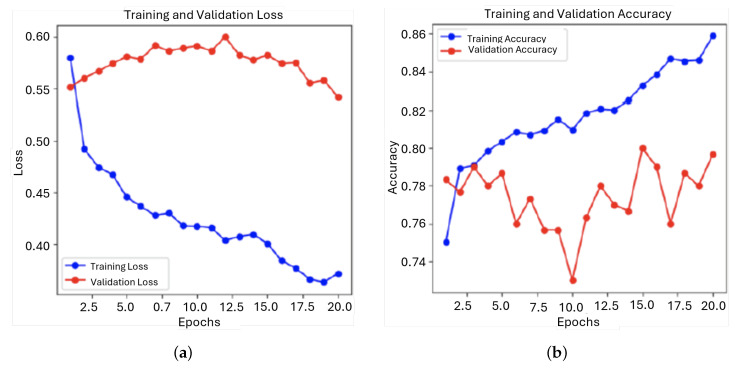
Evolution of the loss function and the accuracy during training and validation for the first model: (**a**) Loss; (**b**) Accuracy.

During training, the model exhibited a consistent decrease in training loss, indicating that it was learning from the data. However, the validation loss showed initial improvement followed by fluctuations and a slight increase, suggesting overfitting. This was further demonstrated by the disparity between the steady improvement in training accuracy and the considerable variability in validation accuracy, highlighting the model’s difficulties in generalizing to new data (Figure 11).

**Figure 12:**
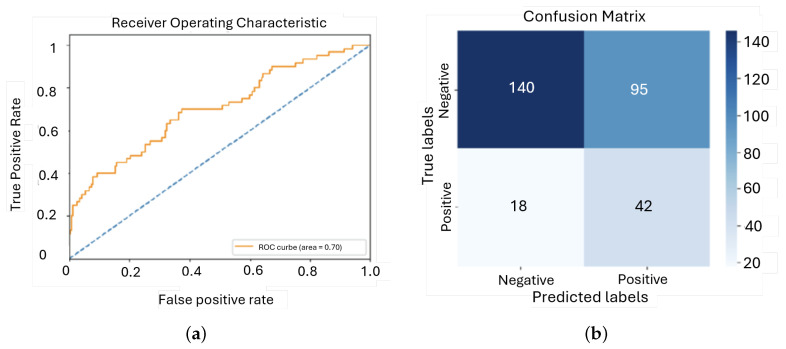
ROC curve and confusion matrix for first model: (**a**) ROC; (**b**) Confusion matrix.

The model achieved an AUC of approximately 0.70, which indicates that its performance was significantly better than random guessing, but still lacking in precision. This level of performance suggests that the model can distinguish between the classes to some extent, but it is not reliable enough for high-stakes clinical applications where maximum accuracy is critical (Figure 12).

While the initial CNN model demonstrated the potential to identify patterns related to cardiac murmurs in the training data, it struggled with generalization and consistency when applied to validation data. This highlights the need for further model refinement, including the use of advanced data augmentation techniques, optimization of learning rates, and the incorporation of early stopping to prevent overfitting. Future improvements will focus on enhancing the model’s robustness and accuracy, making it more reliable for clinical deployment.

### 3.2. Balanced Model

**Figure 13:**
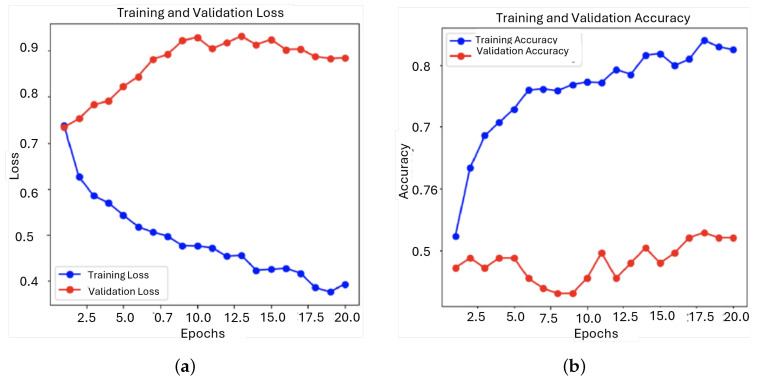
Evolution of the loss function and the accuracy during training and validation for the second model: (**a**) Loss; (**b**) Accuracy.

After analyzing the initial results, an important adjustment was made to the model by balancing the dataset through undersampling of the majority class. This involved reducing the number of ‘murmur absent’ examples to match the ‘murmur present’ class, resulting in a more balanced dataset with 493 instances of ‘murmur present’ and 473 instances of ‘murmur absent’ to whom evolution of the loss and accuracy can be seen in Figure 13.

**Figure 14:**
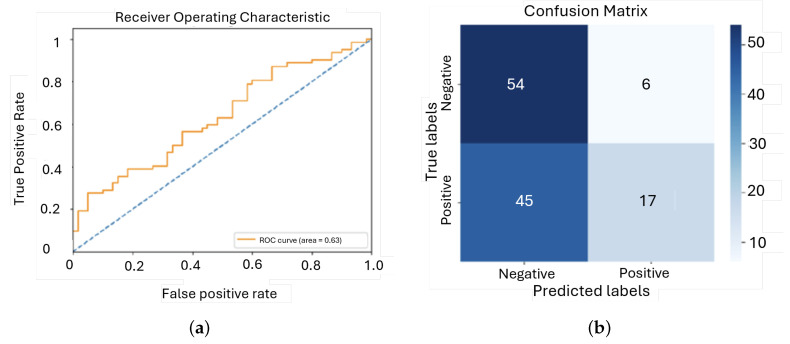
ROC curve and confusion matrix for second model: (**a**) ROC; (**b**) Confusion matrix.

The training loss and accuracy for the balanced model indicate that the model learns well from the training data. However, the validation loss starts at around 0.7, increases to about 0.9 within the first ten epochs, and then slightly decreases, stabilizing at about 0.85, as Figure 14 shows. This could mean that the initial rise and subsequent stabilization imply difficulty in generalizing to the validation set, which could be an overfitting issue. The validation accuracy remains relatively constant, oscillating between 0.5 and 0.6 with a great dispersion among the epochs.

The ROC curve for this model shows moderate performance, with an AUC of 0.63, indicating that while the model is better than random guessing, there is still significant room for improvement. The confusion matrix reveals that the model mispredicts many positive cases, with 45 false negatives. This suggests that the model struggles to correctly identify positive cases, which is critical for clinical applications.

The fact that undersampling did not produce optimal results points to the need for alternative strategies, such as oversampling or data augmentation, to improve model performance and address these issues.

### 3.3. Improved Model

**Figure 15:**
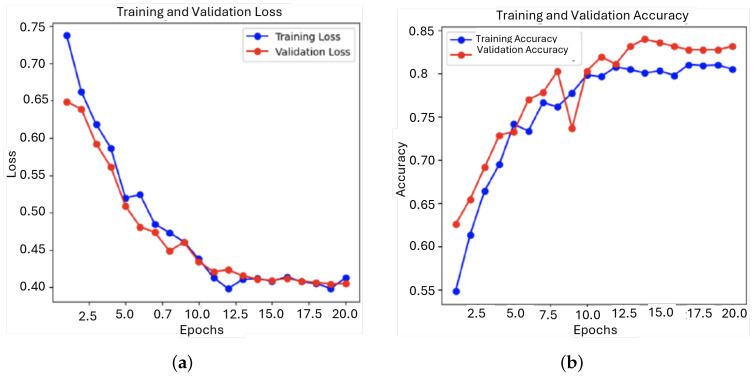
Evolution of the loss function and the accuracy during training and validation for the third model: (**a**) Loss; (**b**) Accuracy.

In response to the limitations of the previous models, several key adjustments were made for this next iteration. The architecture was updated to include three convolutional layers combined with an LSTM layer, thus making it a CRNN. This adaptation was done to take better temporal and spatial features. To address the class imbalance observed in earlier models, the dataset was augmented and oversampled, resulting in a more balanced distribution, with 997 instances of ‘murmur present’ and 1023 instances of ‘murmur absent’. This helped reduce bias toward the majority class. To improve model performance, several optimization techniques were applied. A dynamic learning rate scheduler was introduced to adjust the learning rate during training, allowing for more efficient convergence and reducing the risk of overfitting. Additionally, early stopping, dropout, and L2 regularization were implemented to further prevent overfitting. The evolution of this experiment training can be seen in Figure 15.

It was apparent that this model learns from the training data quite effectively as the prediction error reduced in steps. The accuracy during training grew significantly within the first five epochs from about 0.55 to over 0.75 and further grew rather slowly to about 0.85, which would signify that the model enhances greatly in the ability of accurate classification on training data. It started at around 0.65 and consistently went down to about 0.41, reflecting the trend in training loss. This implies very good generalization of the model to the validation set. Likewise, the accuracy of the model on the validation set has increased from approximately 0.60 to over 0.80, again indicating good generalization.

The loss graph showed a sharp decrease at the beginning that stabilized, which is typical for a well-trained model. The accuracy graph converged between training and validation accuracy, which means the model is well-balanced.

**Figure 16:**
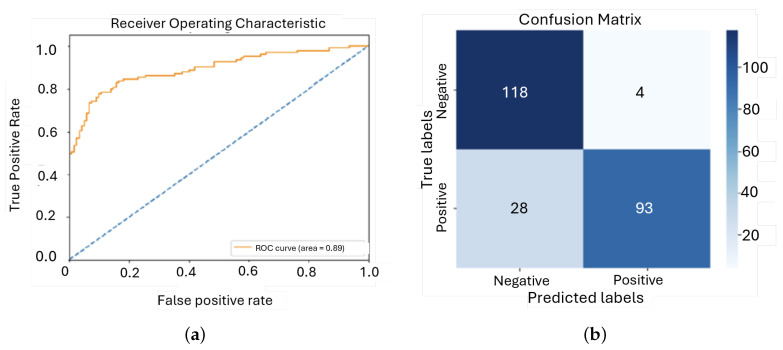
ROC curve and confusion matrix for third model: (**a**) ROC; (**b**) Confusion matrix.

The model performed well, as the metrics shows in Figure 16, highlighting a consistent decrease in loss with a steady increase in accuracy on both training and validation sets. The close values of loss and accuracy for both the training and validation phases suggest that this model generalizes well and does not suffer overfitting. The stabilization at the end of training shows stability in the model, an important trait in practical uses.

These improvements indicate that the integration of CRNN architecture and advanced training techniques has significantly enhanced the model’s performance, making it robust and reliable for detecting cardiac murmurs in practical settings.

### 3.4. Fine-Tuned CRNN

Given the satisfying results from the improved model, it was decided to proceed with this line and apply fine-tuning techniques to further optimize the performance of the model. This involved slight changes in the hyperparameters and architecture but not touching the core of the model, so that it acquires fine-tuned performance adaptability to the dataset.

**Figure 17:**
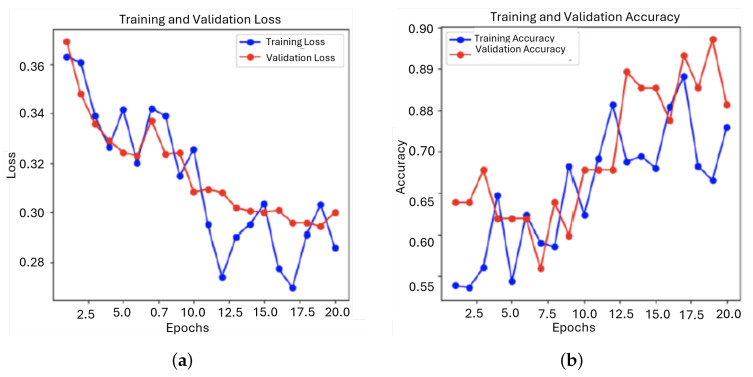
Evolution of the loss function and the accuracy during training and validation for the first model: (**a**) Loss; (**b**) Accuracy.

The fine-tuning process followed brought about more visible improvements in model performance, as evidenced by decreased loss and increased accuracy (Figure 17). By further fine-tuning the hyperparameters and retraining the model, its predictive performance was optimized.

Training and validation loss gradually decreased, indicating that the model was learning efficiently and correctly adjusting parameters for minimal error.

**Figure 18:**
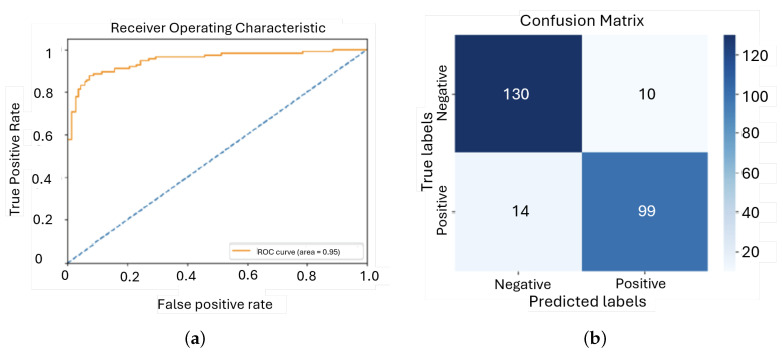
ROC curve and confusion matrix for the fourth model: (**a**) ROC; (**b**) Confusion matrix.

Training and validation accuracy gradually increased, with significant improvements in model performance (Figure 18).

The ROC curve is situated on the top left corner, which suggests that this model performs very well. Also, an AUC value of 0.95 shows that the model exhibits excellent discrimination power between the positive and negative classes. The overall accuracy was 90.5%, indicating that the model had great capability to correctly classify the majority of cases. The model had high precision; when the model predicted a positive class, it was correct 89% of the time, hence it effectively avoided false positives. These results show that fine-tuning is an effective step in model optimization, strengthening the model’s generalization and accuracy across diverse datasets. This approach ensures that not only does the model learn the hidden patterns of the training data well but also extrapolates this knowledge to new, unseen data—a very important aspect for a real-world application.

## 4. Conclusions

This study has demonstrated the potential of artificial intelligence, specifically convolutional recurrent neural networks, to revolutionize the field of non-invasive cardiovascular diagnostics. By developing a robust CRNN model capable of detecting heart murmurs with impressive accuracy, precision, and recall, it has paved the way for a paradigm shift in cardiac care.

The results presented in this work are truly remarkable. The CRNN model achieved an accuracy of 90.5%, precision of 89%, and recall of 87%—performance metrics that far surpass traditional diagnostic methods reliant on physical auscultation and advanced imaging techniques. These findings underscore the model’s ability to identify complex patterns in heart sound recordings that may not be audible to the human ear, underscoring the power of AI-driven analysis.

To further emphasize the novel contributions of this work, a comparative analysis with existing literature was conducted. For instance, the Dual Bayesian ResNet model presented by Benjamin Walker [30] segments heart recordings into overlapping log mel spectrograms for classification and achieved a weighted accuracy of 77.1% on the hidden test set for murmur classification. The superior performance metrics of our CRNN model underscore its effectiveness and potential for clinical application.

The integration of convolutional recurrent neural networks into clinical practice holds significant promise for early intervention and improved patient outcomes. CRNN models can be seamlessly integrated with existing diagnostic systems, such as electronic health record (EHR) systems, to provide comprehensive access to patient data and diagnostic results. APIs enable CRNN models to communicate with other diagnostic tools, facilitating data exchange and a holistic approach to patient care. User-friendly interfaces for clinicians can enhance the adoption and usability of the technology, visualizing results and providing actionable insights.

CRNN models process data in real time with minimal latency, depending on the model’s complexity and available computational resources. Optimizing the model and utilizing high-performance hardware can further reduce latency, ensuring efficient handling of large data volumes. Techniques such as model pruning, quantization, and distillation can enhance the models’ speed and efficiency, making them suitable for real-time applications.

In conclusion, this research represents a significant stride forward in the quest to enhance non-invasive cardiac care. By seamlessly blending medical expertise with cutting-edge machine learning, the study has demonstrated the transformative power of AI in addressing longstanding challenges and improving the lives of patients worldwide.

## Figures and Tables

**Table 1:** The metric values for the developed models.

Model	Number of Samples			Metrics’ Values		
Version	Train	Validation	Test	Loss	acc.	AUC.
Baseline model	2096	262	262	0.55	0.78	0.70
Balanced model	1942	242	242	0.88	0.50	0.63
Improved model	1942	242	242	0.4	0.84	0.89
Fine-Tuned model	1942	242	242	0.30	0.905	0.85

## Data Availability

The database used as input is called The CirCor DigiScope Phonocardiogram Dataset Version: 1.0.3, available at https://physionet.org/content/circor-heart-sound/1.0.3/.
